# 

**Published:** 1896-05

**Authors:** 


### CONTENTS.

COMMUNICATIONS.

Ethics ..... 209
The Literary Side of our Profession ..... 216
The Good and the Objectional Qualities of Oxyphosphate of Zinc as a Filling Material ..... 220
What Difficulties does the Dentist Encounter from the Presence of Secondary Formations? ..... 222
Progressive Calcification ..... 224
A Suggestion on the Origin of Some Cases of Pyorrhœa Alveolaris ..... 230
A Suggestion ..... 234
The Bridge Case—Decision of the Court ..... 237

SELECTIONS.

Hygiene of the Face ..... 240
The Sanitation of Work-shops and Public Conveyances ..... 241
Camphor for Ether Collapse ..... 246
Resorption of the Salts of Iron ..... 247
A Superfluous Detail ..... 247

NOTICES.

American Medical Association—Section on Dental and Oral Surgery ..... 248
New York State Dental Society ..... 248
The Kentucky State Dental Association ..... 249
Maryland State Dental Association and Washington City Dental Society ..... 249

EDITORIAL.

National Library and Museum—Dental ..... 251
Mississippi Valley Dental Society ..... 254
Popular Education in Dentistry ..... 255
Biographical Sketch of Dental Pathology and Practice ..... 256
Biographical ..... 257
Biographical ..... 259

